# Lung Cancer Detection Using Bayesian Networks: A Retrospective Development and Validation Study on a Danish Population of High‐Risk Individuals

**DOI:** 10.1002/cam4.70458

**Published:** 2025-01-31

**Authors:** Margrethe Bang Henriksen, Florian Van Daalen, Leonard Wee, Torben Frøstrup Hansen, Lars Henrik Jensen, Claus Lohman Brasen, Ole Hilberg, Inigo Bermejo

**Affiliations:** ^1^ Department of Oncology Vejle University Hospital Vejle Denmark; ^2^ Institute of Regional Health Research University of Southern Denmark Odense Denmark; ^3^ Department of Radiation Oncology (MAASTRO) GROW School for Oncology and Reproduction Maastricht University Medical Centre+ Maastricht the Netherlands; ^4^ Department of Biochemistry and Immunology Vejle University Hospital Vejle Denmark; ^5^ Department of Internal Medicine Vejle University Hospital Vejle Denmark; ^6^ Data Science Institute (DSI) Hasselt University Hasselt Belgium

## Abstract

**Background:**

Lung cancer (LC) is the top cause of cancer deaths globally, prompting many countries to adopt LC screening programs. While screening typically relies on age and smoking intensity, more efficient risk models exist. We devised a Bayesian network (BN) for LC detection, testing its resilience with varying degrees of missing data and comparing it to a prior machine learning (ML) model.

**Methods:**

We analyzed data from 9940 patients referred for LC assessment in Southern Denmark from 2009 to 2018. Variables included age, sex, smoking, and lab results. Our experiments varied missing data (0%–30%), BN structure (expert‐based vs. data‐driven), and discretization method (standard vs. data‐driven).

**Results:**

Across all missing data levels, area under the curve (AUC) remained steady, ranging from 0.737 to 0.757, compared to the ML model's AUC of 0.77. BN structure and discretization method had minimal impact on performance. BNs were well calibrated overall, with a net benefit in decision curve analysis when predicted risk exceeded 5%.

**Conclusion:**

BN models showed resilience with up to 30% missing values. Moreover, these BNs exhibited similar performance, calibration, and clinical utility compared to the machine learning model developed using the same dataset. Considering their effectiveness in handling missing data, BNs emerge as a relevant method for the development of future lung cancer detection models.

## Graphical abstract

We employed Bayesian networks to predict lung cancer risk within a high‐risk Danish cohort. Regardless of the extent of missing data, Bayesian networks yielded consistent results, suggesting potential for future lung cancer detection models.

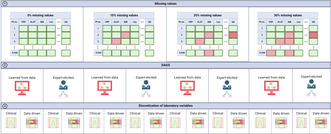

## 1 Introduction

Lung cancer (LC) is the leading cause of cancer‐related deaths globally, accounting for 18% of all cancer deaths in 2020, with 1.8 million new deaths reported [1]. LC is often detected in advanced stages, limiting treatment options [2]. However, early‐stage LC offers a more favorable prognosis, since these patients are often eligible for curative treatment [3]. Consequently, multiple screening initiatives have been implemented over the past decade, aiming to enhance LC survival rates by promoting early‐stage detection [4]. Results from the Dutch–Belgian NELSON trial have demonstrated a 25% reduction in LC mortality through low‐dose computed tomography (CT) over a 10‐year follow‐up period [5]. Similarly, the American NLST trial has exhibited a 20% decrease in mortality after a median follow‐up of 6.5 years [6]. Building upon these findings, the U.S. Preventive Services Task Force (USPSTF) advocates for annual LC screening using low‐dose CT for high‐risk individuals, determined by age and smoking history [7].

Despite the encouraging decrease in mortality, concerns have been raised about the underdiagnosis resulting from the adoption of narrow, fixed selection criteria. For instance, adhering to the USPSTF screening criteria would only detect 68% of LC patients in the United States [8]. On the other hand, a high false‐positive rate poses disadvantages such as resource consumption, increased patient anxiety, and the need for invasive procedures during follow‐up evaluations.

Over the past decade, various risk prediction models, such as PLCOm2012, have emerged, demonstrating higher sensitivity than the USPSTF screening criteria [9]. External validations of these models have led to their inclusion in the National Comprehensive Cancer Network guidelines [10, 11, 12]. Most risk models rely on established risk factors or novel biomarkers, analyzed through traditional regression analyses. However, the recent surge in interest in machine learning (ML) and deep neural networks, capable of handling complex data, has paved the way for more advanced models [13, 14].

While standard blood sample analyses are commonplace, cost‐effective, and easily obtainable, their application in early LC identification has been limited [15, 16]. The XGBoost algorithm developed by Medial Early Sign (“MES” model) outperformed the PLCO2012 model, incorporating laboratory results and smoking history [15]. Based on the same type of variables from a Danish high‐risk cohort [17], the Dynamic Ensemble Selection (DES) ML model was recently developed by Flyckt et al. [18]. The DES model demonstrated moderate performance, although not surpassing the PLCO2012 model [18]. This study revisits the Danish high‐risk population, employing Bayesian networks (BNs) for LC classification.

BNs are probabilistic graphical models that represent variables and their conditional dependencies within a directed acyclic graph (DAG). These models have gained widespread application in the realms of AI and healthcare, offering distinct advantages over ML models [19, 20, 21, 22]. They excel in incorporating both causal as well as associative relationships, facilitating the integration of data with expert knowledge [23], and functioning as decision support tools [22, 24, 25]. Crucially, BNs are able to handle missing data through probabilistic inference, making them particularly valuable in healthcare and for screening purposes, where data may be hard to obtain [26]. BNs can be trained on larger datasets without the explicit need to filter out or impute missing records. Moreover, BNs enable the combination of data from different hospitals even when there are variations in data collection protocols, which results in different sets of data being collected at each hospital. The integration of expert knowledge, coupled with the fact that BNs are relatively easy to understand for medical professionals without an AI background, enhances its suitability for adoption in clinical settings. This primary motivation underscores our exploration of the potential applications of BNs.

BNs have been extensively utilized in LC research, with the predominant focus of these studies being on the prediction of patient survival and supporting treatment decisions [27, 28, 29]. However, their application in LC detection or screening is less common. A dynamic BN model was developed using a high‐risk cohort from the NLST dataset to predict LC from clinical and demographic data, outperforming logistic regression models and being comparable to human experts [30]. In a subsequent study on the same dataset, BN and ML approaches were combined, which resulted in improved performance compared to experts [31]. None of these studies have applied BNs to standard laboratory data, nor have they, to our knowledge, experimentally tested performance by gradually increasing the rate of missing data. Moreover, the majority of studies typically employ a single methodology for reporting, thereby complicating direct comparisons. A distinctive aspect of our approach is that we develop BN models using the same dataset as previously used for ML models, enabling direct performance comparisons.

The paper's objective is to compare different BN structures' performance with the DES model, utilizing the same population and dataset. Additionally, the study seeks to investigate performance variations when introducing varying degrees of missing values.

In this article, we follow the TRIPOD reporting guidelines for the development and validation of prediction models [32].

## 2 Methods

### 2.1 Study Cohort and Data Collection

**FIGURE 1:**
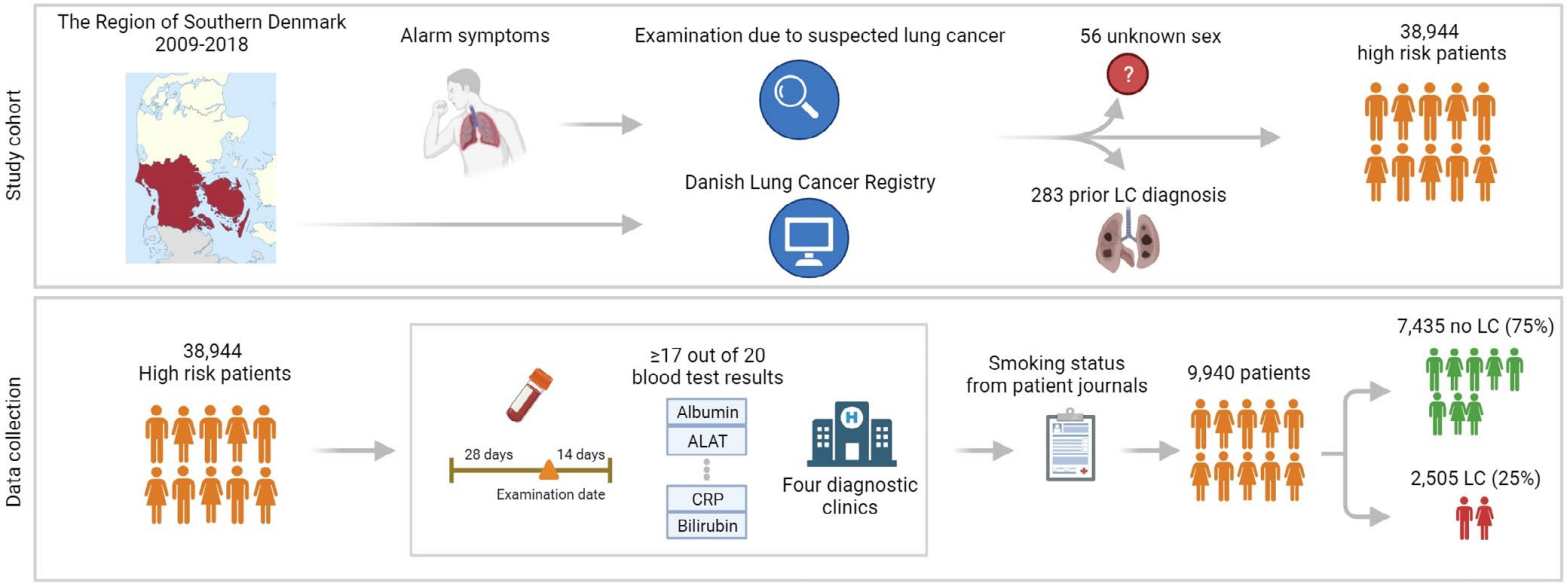
Overview of the study cohort and data collection involving all patients in the region of Southern Denmark, examined on suspicion of LC over the period 2009–2018. ALAT, alanine transaminase; CRP, C‐reactive protein. Created with Biorender.com.

The dataset employed in this study originated from a previously defined cohort and is outlined in Figure 1 [17, 18]. It includes all patients examined on suspicion of LC between January 1, 2009, and December 31, 2018, within the Region of Southern Denmark. Data were sourced from the regional data warehouse and integrated with information from the Danish Lung Cancer Registry. Despite the capability of BNs to manage missing data, we implemented identical inclusion criteria and filtering as the high‐risk study cohort mentioned earlier [18]. This approach ensures a comparative analysis of performance on the identical population. After the exclusion of 56 individuals without information on sex and 283 because of a prior LC diagnosis, previous blood sample results were collected within an interval spanning 28 days before the examination date to 14 days after. To be included, patients needed to have results for at least 17 out of 20 relevant blood sample analyses, allowing a maximum of three missing variables per patient. Additionally, only data from the four LC fast‐track diagnostic clinics were considered. Information on smoking status was derived from manual annotation of free text in the electronic health records.

Applying these criteria led to a final dataset of 9940 patients, of whom 7435 were diagnosed with LC (85%) and 2505 were not (25%). Sex and smoking status were noted as discrete variables (never smoker vs. active/former smoker), while age and results for the 20 laboratory analyses were continuous variables. Within the cohort of included patients, approximately 3% of the data were missing, and no imputation was performed.

### 2.2 Experimental Setup

**FIGURE 2:**
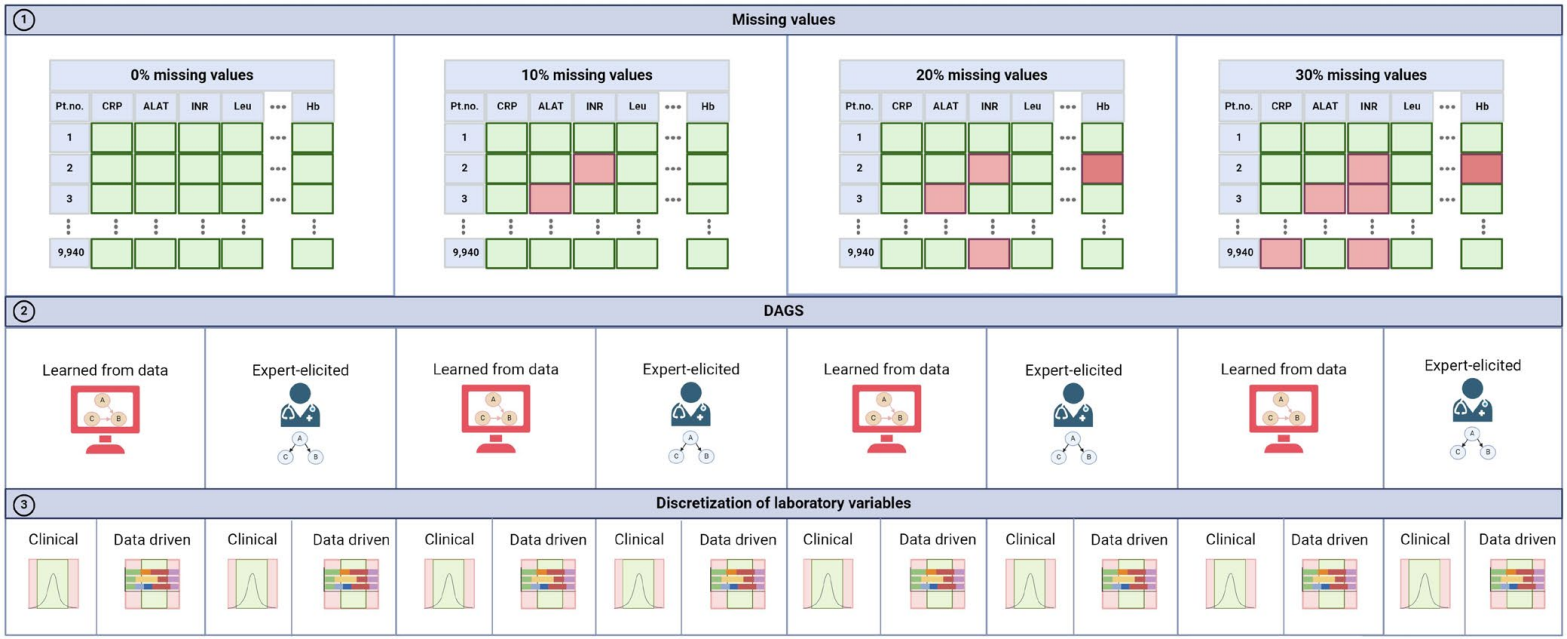
The experimental setup involving varying degrees of missing values, comparing expert‐elicited DAGs with DAGs learnt from data, and utilizing both standard and data‐driven discretization. These procedures led to the development of a total of 16 different models from the dataset. ALAT, alanine transaminase; CRP, C‐reactive protein; Hb, hemoglobin; INR, International normalized ratio; Pt.no., patient number. Created with Biorender.com.

The experimental setup was based on three different subtypes of analyses, depicted in Figure 2.

#### 2.2.1 Different Degrees of Missing Values

To simulate real‐world scenarios where data might be incomplete, we introduced missing values into the dataset by randomly deleting existing values. This process was conducted systematically to create varying levels of missing data: 0%, 10%, 20%, and 30%. Missing values were randomly generated, ensuring they are missing completely at random.

#### 2.2.2 DAGs Learnt From Data Versus Expert‐Elicited

BNs were trained using a DAG created by three clinicians with expertise in oncology, pulmonology, and biochemistry (referred to as the “expert‐elicited DAG”). Experts reviewed the current dataset and created a DAG to depict causal or probabilistic relationships from a clinical perspective. Inconsistencies were addressed, leading to a consensus on a unified DAG version. The DAG underwent multiple revisions based on expert feedback and insights, resulting in a final version that integrates diverse domain knowledge.

Simultaneously, a DAG referred to as the “DAG learned from data” was learnt from data using the K2 algorithm, which is a fundamental method used in probabilistic graphical models like BNs [33]. Named after the mountain K2, it compares the challenges of learning BNs from data to climbing a tall mountain. The K2 algorithm was used for structure learning, assuming complete data, replacing missing continuous attributes with the mean, and missing discrete attributes with the mode. The structure of the K2‐DAG was built step by step, by adding one connection at a time, assessing each added connection's predictive performance to form the most representative DAG of probabilistic relationships among variables. We conducted two analyses: one starting with a straightforward variable‐outcome model (LC) and another without initial assumptions. The K2 algorithm was tested with varying limits on parent nodes per variable in the DAG, from 1 to 10. The best structure was determined based on its AUC (area under the ROC curve), validated through 10‐fold cross‐validation.

In the parameter learning phase of both the expert‐elicited DAG and the DAG learned from data, we determined specific probabilities (or conditional dependencies) established between the variables using the expectation maximization algorithm. This technique handles missing data by estimating missing values in the expectation step and refining the model in the maximization step, ultimately enhancing model performance [34].

#### 2.2.3 Clinical Versus Data‐Driven Discretization of Laboratory Variables

We compared clinical discretization, which uses standard reference intervals, with a data‐driven approach based on the minimum description length (MDL) strategy [35]. The MDL strategy seeks to identify the model that minimizes the information needed to describe the data. It involves selecting the optimal number of bins for continuous laboratory variables, striking the best trade‐off between model simplicity and accuracy in representing the underlying data distribution. Table 1 displays the categories and boundaries for both methods across 20 laboratory analyses with 0% missing values. Table S1 extends these comparisons to other missing value levels, noting consistent attributes across levels.

**TABLE 1:** Comparison between clinical discretization values derived from clinical guidelines' reference intervals and data‐driven discretization values using minimum description length, illustrated for data with 0% missing values.

	Clinical discretization from reference intervals (95% CI)	Data‐driven discretization based on minimum description length, 0% missing values^a^
P‐ALAT, U/L	Male: 10–70, Female: 10–45	15.5–24.5
P‐Albumin, g/L	34–45	< 41.5
P‐Amylase (pancreatic), U/L	10–65	ALL
P‐Alkaline phosphatase	35–105	< 84.5
B‐Basophils, 10^9^/L	< 0.02	< 0.05
P‐Bilirubin‐total, μmol/L	5–25	< 7.5
P‐CRP, mg/L	< 6	3.05–13.50
Total Calcium, mmol/L	2.15–2.51	< 2.40
B‐Eosinophils, 10^9^/L	< 0.05	< 0.10
B‐Hemoglobin, mmol/L	Male: 8.3–10.5, Female: 7.3–9.5	8.05–9.25
P‐INR	< 1.2	< 0.80
P‐Potassium, mmol/L	3.5–4.4	ALL
P‐Creatinine, mmol/L	Male: 60–105, Female: 45–90	< 68.5
P‐LDH, U/L	115–255	187.5–212.5−275.5−417.5
B‐Leucocytes, 10^9^/L	3.5–8.8	6.2–7.9−11.2
B‐Lymphocytes, 10^9^/L	1.0–4.0	ALL
B‐Monocytes, 10^9^/L	0.2–0.8	< 0.7
P‐Sodium, mmol/L	137–145	135.5–138.5
B‐Neutrophils, 10^9^/L	1.5–7.5	3.5–5.1−7.0
B‐Platelets, 10^9^/L	Male: 145–350, Female: 165–390	251.5–342.5

*Note:* The numbers in each column indicate the threshold values for binning. For example, the data‐driven discretization for ALAT yields three bins: (−∞; 15.5), [15.5; 24.5), and [24.5; ∞). Likewise, LDH's data‐driven discretization involves four cutoff points, leading to five bins. “All” denotes the absence of cutoff values, resulting in a single bin encompassing all data points.

Abbreviations: ALAT, alanine transaminase; CRP, C‐reactive protein; INR, International normalized ratio; LDH, lactate dehydrogenase.

^a^ Results for other degrees of missing values than 0% are displayed in Data S1.

### 2.3 Statistical Analyses

Baseline characteristics were described using the median and interquartile range (IQR) for continuous variables, and number and percentage for categorical variables. The validation of the experiments involved employing a 10‐fold cross‐validation technique, with 95% confidence intervals (CI) provided for the AUC. Discrimination was assessed through the AUCs, and the true positive rate (TPR/sensitivity) and true negative rate (TNR, specificity) were evaluated at the default probability cut‐off of 0.5. A comparative analysis of the TPR against other relevant models was performed while maintaining a fixed TNR of 95%. Calibration was examined by comparing predicted and observed risk across the overall cohort (mean calibration) and various stages of LC (stratified calibration). The clinical utility was assessed using decision curve analysis, where the net benefit was reported at different probability cutoffs. The net benefit of employing the selected BN model was compared to the strategy of assessing all patients for LC examination and the strategy of assessing no patients [36].

All experiments were conducted using the WEKA framework version 3.8 [37].

### 2.4 Ethics Statement

The study was conducted in accordance with the Declaration of Helsinki (as revised in 2013) and approved by the Danish Data Protection Agency (19/30673, 06‐12‐2020) and the Danish Patient Safety Authority (3‐3013‐3132/1, 03‐30‐2020). Individual consent for this retrospective analysis was waived.

## 3 Results

### 3.1 Baseline Characteristics

Table 2 illustrates the baseline characteristics of 9940 patients, categorized into 2505 with LC and 7435 without LC. The median age for LC patients was 74 years (IQR 68–80), contrasting with 71 years (IQR 59–79) in the non‐LC group. Females constituted 52.1% of the LC group, while the non‐LC group had 44.0% females. Smoking prevalence was notably higher among LC patients, with 92.2% identified as smokers, in contrast to 69.2% among non‐LC patients. Although differences in laboratory measures were generally subtle, median values for both LC and non‐LC groups mostly fell within the standard reference interval, as depicted in Table 1.

**TABLE 2:** Baseline characteristics for the lung cancer (LC) cohort and non‐LC cohort. Continuous measures are displayed in medians with interquartile range, and categorical values in counts and percentages.

	LC (*n* = 2505)	Non‐LC (*n* = 7435)
Age, years	74 (68–80)	71 (59–79)
Sex		
Female	1304 (52.1%)	3273 (44.0%)
Male	1201 (47.9%)	4162 (56.0%)
Smoking status		
Never smoker	196 (7.8%)	2288 (30.8%)
Former/current smoker	2309 (92.2%)	5147 (69.2%)
Blood sample analyses		
P‐ALAT, U/L	19 (14–26)	22 (16–31)
P‐Albumin, g/L	42 (40–45)	43 (41–45)
P‐Amylase (pancreatic), U/L	25 (19–34)	25 (18–33)
P‐Alkaline phosphatase	81 (67–99)	74 (62–91)
B‐Basophils, 10^9^/L	0.05 (0.02–0.06)	0.04 (0.02–0.06)
P‐Bilirubin‐total, μmol/L	7 (5–9)	7 (5–10)
P‐CRP, mg/L	7.0 (2.3–22.0)	3.4 (1.4–9.3)
Total calcium, mmol/L	2.38 (2.31–2.45)	2.34 (2.28–2.41)
B‐Eosinophils, 10^9^/L	0.14 (0.08–0.24)	0.17 (0.10–0.28)
B‐Hemoglobin, mmol/L	8.5 (7.8–9.1)	8.7 (8.1–9.3)
P‐INR	1.00 (0.94–1.08)	1.00 (0.95–1.10)
P‐Potassium, mmol/L	4.0 (3.8–4.3)	4.0 (3.8–4.3)
P‐Creatinine, mmol/L	72 (61–87)	76 (64–90)
P‐LDH, U/L	209 (182–246)	192 (169–220)
B‐Leucocytes, 10^9^/L	8.80 (7.29–10.70)	7.62 (6.20–9.38)
B‐Lymphocytes, 10^9^/L	1.79 (1.37–2.34)	1.84 (1.40–2.37)
B‐Monocytes, 10^9^/L	0.73 (0.57–0.93)	0.65 (0.51–0.83)
P‐Sodium, mmol/L	139 (136–141)	140 (138–142)
B‐Neutrophils, 10^9^/L	5.77 (4.52–7.42)	4.66 (3.54–6.11)
B‐Platelets, 10^9^/L	301 (243–378)	271 (224–331)
LC stage		
Stage I	799 (31.9%)	NA
Stage II	286 (11.4%)	NA
Stage III	592 (23.6%)	NA
Stage IV	792 (31.6%)	NA
Unknown	36 (1.4%)	NA

Abbreviations: ALAT, alanine transaminase; CRP, C‐reactive protein; INR, International normalized ratio; LDH, lactate dehydrogenase.

### 3.2 Model Evaluation

The combination of the three subtypes of analyses (four degrees of missing values, two types of DAGs, and two types of discretization strategies) resulted in 16 different models with performance metrics. We compared the results to the previously presented DES model, achieving an AUC of 0.77 and a TPR of 24% at a TNR of 95%.

Table 3 displays the AUC, TPR, and TNR alongside the 95% CI for all 16 models. In general, AUCs were consistent across the different levels of missing values, ranging from a minimum of 0.737 (95% CI: 0.711–0.763) for Model 11 to a maximum of 0.757 (95%CI: 0.729–0.783) for Model 6. Based on the overlapping confidence intervals, we concluded that there was no statistically significant difference in AUCs among the models. This conclusion held true when comparing both DAG structures and discretization strategies, as confidence intervals generally overlapped across all levels of missing values.

**TABLE 3:** Evaluation metrics of the 16 models, derived from the combination of the level of missing data, type of DAG, and discretization strategy.

DAG type	DAG learned from data		Expert‐elicited DAG		DAG learned from data		Expert‐elicited DAG	
Discretization	Clinical	Data driven	Clinical	Data driven	Clinical	Data driven	Clinical	Data driven
Missing %	Missing Values: 0%				Missing Values: 10%			
Model no.	Model 1	Model 2	Model 3	Model 4	Model 5	Model 6	Model 7	Model 8
AUC (95% CI)	0.756 (0.729–0.783)	0.756 (0.729–0.783)	0.735 (0.709–0.761)	0.748 (0.722–0.774)	0.751 (0.724–0.778)	0.757 (0.730–0.784)	0.740 (0.714–0.766)	0.746 (0.720–0.772)
TNR (95% CI)^a^	0.889 (0.872–0.905)	0.895 (0.879–0.911)	0.833 (0.818–0.848)	0.875 (0.859–0.890)	0.894 (0.878–0.910)	0.906 (0.890–0.923)	0.848 (0.833–0.864)	0.883 (0.867–0.899)
TPR (95% CI)^a^	0.365 (0.358–0.371)	0.344 (0.338–0.350)	0.444 (0.436–0.452)	0.371 (0.364–0.770)	0.327 (0.321–0.333)	0.314 (0.308–0.319)	0.418 (0.411–0.426)	0.341 (0.334–0.347)
TPR at TNR=0.95	0.202 (0.195–0.209)	0.206 (0.199–0.213)	0.186 (0.179–0.193)	0.203 (0.196–0.210)	0.194 (0.187–0.201)	0.19 (0.183–0.197)	0.174 (0.166–0.181)	0.183 (0.176–0.191)

Missing %	Missing Values: 20%				Missing Values: 30%			
Model no.	Model 9	Model 10	Model 11	Model 12	Model 13	Model 14	Model 15	Model 16
AUC (95% CI)	0.7495 (0.723–0.775)	0.748 (0.722–0.774)	0.737 (0.711–0.763)	0.744 (0.718–0.770)	0.753 (0.726–0.780)	0.748 (0.722–0.774)	0.739 (0.713–0.765)	0.745 (0.719–0.771)
TNR (95% CI)^a^	0.900 (0.883–0.916)	0.908 (0.892–0.924)	0.851 (0.844–0.874)	0.892 (0.867–0.908)	0.924 (0.907–0.941)	0.909 (0.892–0.925)	0.874 (0.858–0.890)	0.904 (0.888–0.920)
TPR (95% CI)^a^	0.303 (0.298–0.308)	0.273 (0.268–0.278)	0.374 (0.367–0.380)	0.305 (0.300–0.310)	0.245 (0.240–0.249)	0.272 (0.267–0.276)	0.340 (0.334–0.346)	0.272 (0.267–0.276)
TPR at TNR = 0.95	0.172 (0.164–0.180)	0.171 (0.163–0.178)	0.156 (0.148–0.164)	0.172 (0.164–0.180)	0.16 (0.152–0.168)	0.172 (0.164–0.179=	0.157 (0.149–0.165)	0.166 (0.158–0.173)

Abbreviations: AUC, area under the receiver operating characteristic (ROC) curve; DAG, directed acyclic graph; TNR, true negative rate; TPR, true positive rate.

^a^ TNR and TPR displayed at a default probability cutoff of 0.5.

**FIGURE 3:**
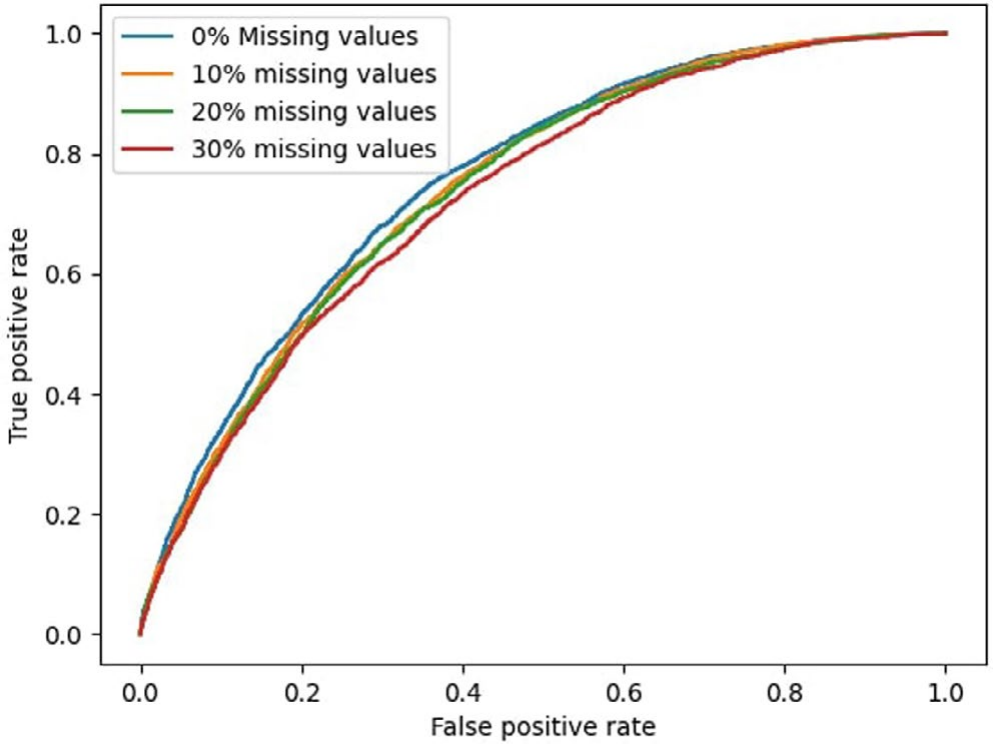
Receiver operating characteristic (ROC) curves of the four levels of missing values. All models were based on DAGs learned from data with data‐driven discretization (model 2, 6, 10, 14 in Table 3).

Figure 3 displays the four ROC curves for models based on DAGs learned from data with data‐driven discretization, categorized according to the level of missing values. ROC curves for the remaining 12 models can be found in the Figures S1–S3. It is evident that the performance of all models is closely aligned. At a fixed TNR of 95%, the maximum TPR is achieved by Model 2, which is 20.6% (95% CI: 19.9%–21.3%). The same model achieves a TPR of 34.4% and TNR of 89.5% at a default probability cut‐off of 50%.

**FIGURE 4:**
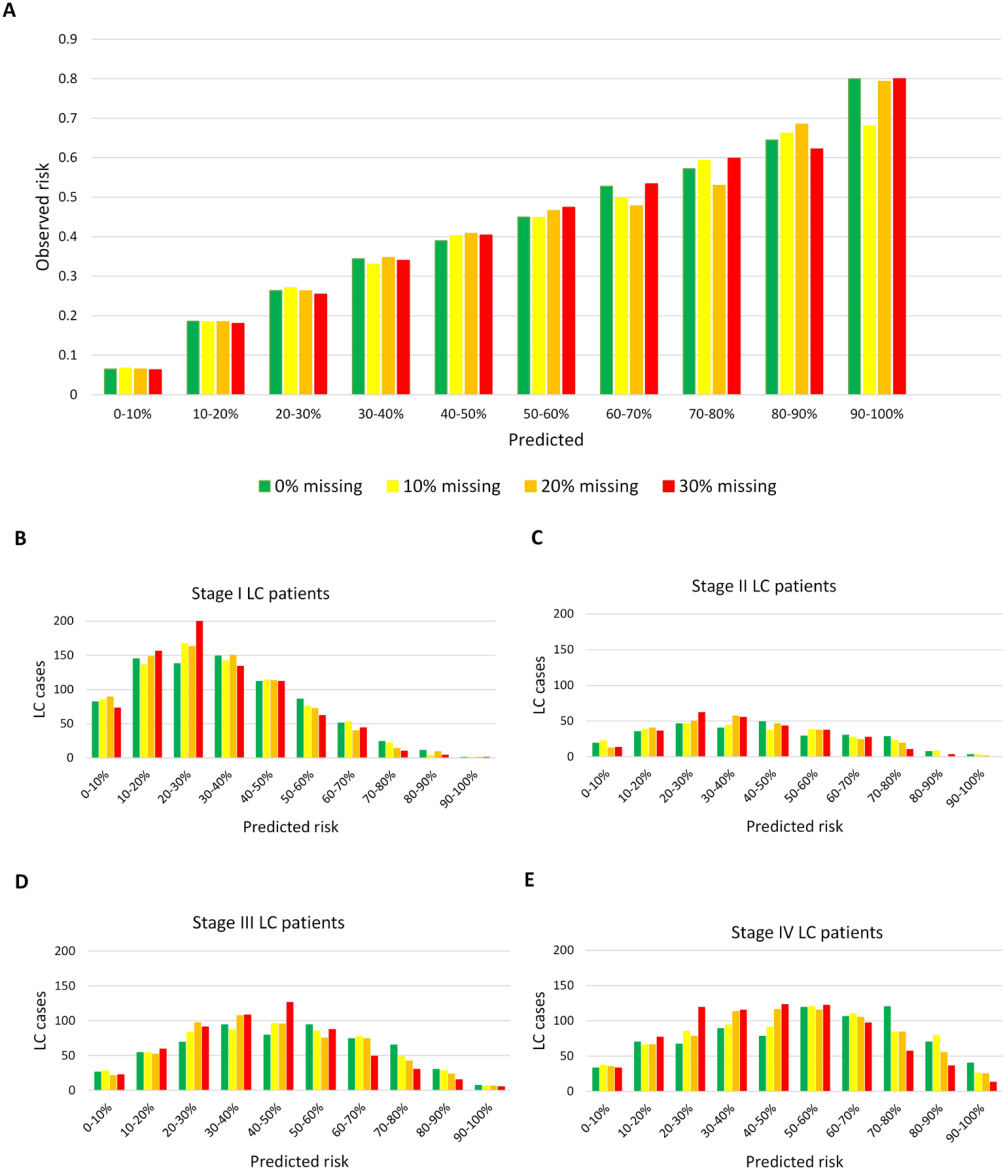
(A) The predicted versus observed risk for all 9940 patients displayed for all four levels of missing values. (B–E) The predicted risk versus the actual number of lung cancer (LC) patients displayed for each Stage I–IV. All graphs are derived from the DAGs learned from data with data‐driven discretization.

The calibration of the model at different risk thresholds is evaluated in Figure 4A. The predicted risk is provided on the x‐axis in bins of 0.1, displayed for all four levels of missing values from the DAGs learned from data with data‐driven discretization (Models 2, 6, 10, and 14). Throughout the plot, regardless of the level of missing values, an increase in predicted risk is followed by an increase in observed risk. For risk intervals ranging between 0% and 40% the model is well calibrated with predicted and observed risks falling inside the same interval. For risk intervals above 40%, the predicted risk generally exceeds that of the observed risk, indicating that the model overestimates the true risk. For instance, among individuals with predicted risk ranging between 50% and 60%, only 40%–50% of these were actually LC patients.

Figure 4B–E displays the predicted risk against the number of true LC patients for each LC stage and for all levels of missing values, using DAGs learned from data with data‐driven discretization. Among the stage I LC patients (31.9%), the majority fell in risk intervals ranging from 10% to 40%. For the remaining patients in stage II (11.4%), III (23.6%), and IV (31.6%), the predicted risk was more evenly distributed, with the majority within the large interval of 20%–70% risk. For all four stages, only a minority of patients fell within the highest risk intervals.

**FIGURE 5:**
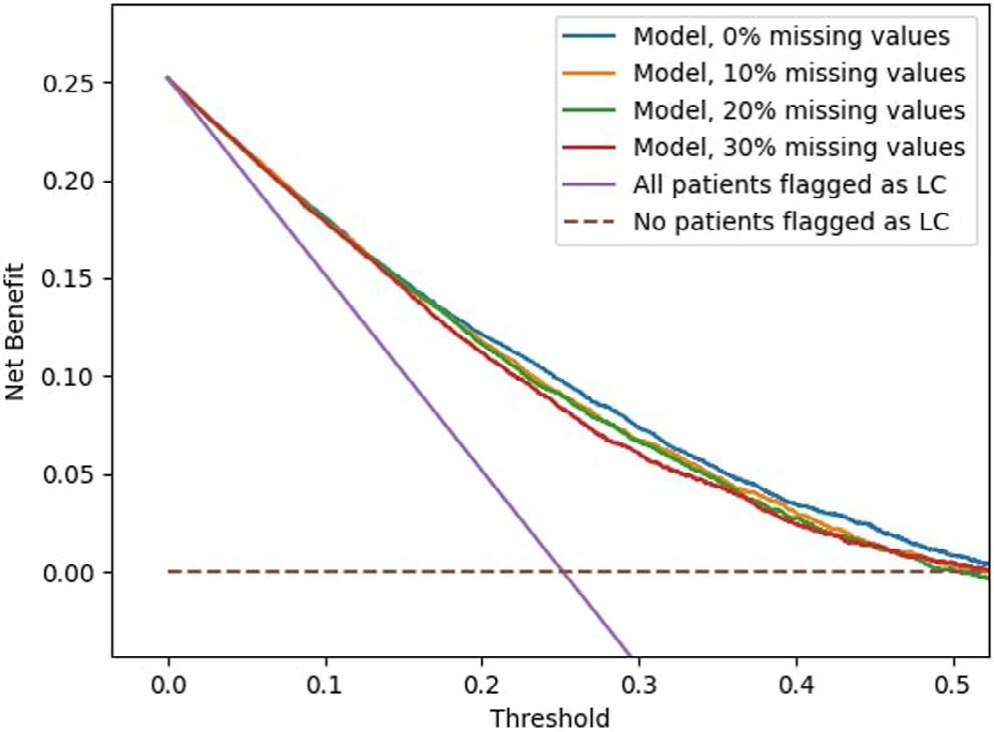
Decision curve analyses displaying the net benefit for all four levels of missing values, compared to the strategy of flagging all (purple) or no patients (brown dashed line) as high risk of lung cancer (LC). All graphs are derived from the DAGs learned from data with data‐driven discretization.

The clinical utility of the models was assessed using decision curve analysis, displayed in Figure 5. The four models displayed represent the four degrees of missing values with DAGs learned from data and data‐driven discretization (Models 2, 6, 10, and 14). The plot displays the trade‐offs between the TPR and FPR of the four models as the threshold probability varies. The net benefit of the four models is compared to the net benefit of the strategy of flagging all patients as individuals who should undergo LC screening (purple line) compared to the strategy of flagging no patients eligible for LC screening (brown dashed line). All four models exhibit similar net benefits at lower risk thresholds, with an enhancement in comparison to flagging all patients when the risk threshold exceeds approximately 5%. This positive net benefit gradually declines until a risk threshold of approximately 50% is reached. From here it levels with the strategy of flagging no patients (brown dashed line). Consequently, the BN models can be considered to include patients in a screening scenario at a minimum threshold of approximately 5% in this population.

**FIGURE 6:**
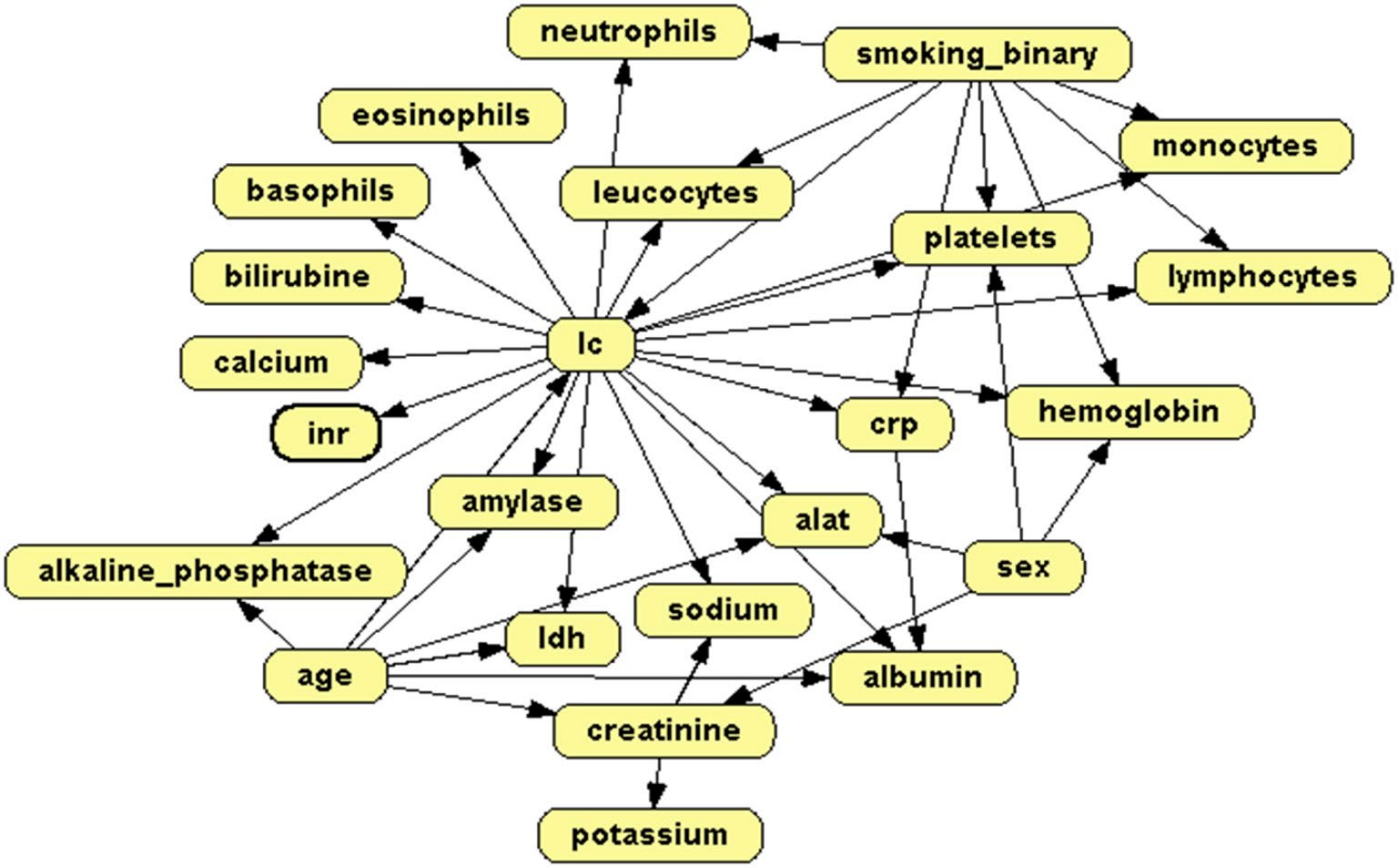
The directed acyclic graph (DAG) provided by experts displaying the interconnections between the outcome lung cancer (LC) and all other variables. Rendered using OPEN Markov [38].

Figure 6 displays the expert‐elicited DAG, which can be compared to the eight DAGs learned from data derived from different degrees of missing values and clinical versus data‐driven discretization strategies (Figure S4). Table 4 makes it easier to compare the DAGs by summarizing all links. Links on the expert‐elicited DAG in the direction from parent node to child node are noted in green (e.g., Age→ALAT), whereas the inversion of these links is noted in orange (e.g., ALAT→Age). Links originating from the DAGs learned from data are listed as numbers inside fields. The specific number refers to the number of DAGs learned from data (out of a total of 8) containing the specific link. For instance, five out of the eight DAGs learned from data display a link between age and ALAT.

**TABLE 4:** Overview of links extracted from the directed acyclic graphs (DAGs) depicted in Figure 6.

Parental node																									
Child node		Age	ALAT	Albumin	Alkaline phosphatase	Amylase	Basophils	Bilirubine	Calcium	Creatinine	CRP	Eosinophils	Hemoglobin	INR	LC	LDH	Leucocytes	Lymphocytes	Monocytes	Neutrophils	Platelets	Potassium	Sex	Smoking	Sodium
	Age														8										
	ALAT	5													8										
	Albumin	8	6												8										
	Alkaline phosphatase		5	8											8										
	Amylase			4											8										
	Basophils			1	4										7										
	Bilirubine		3	6											8										
	Calcium			7		4									7										
	Creatinine	6		2				3							8										
	CRP	1		7	4				4						8										
	Eosinophils						4	1		7					8										
	Hemoglobin		4	8						1	4				7										
	INR											1	4		7										
	LC																								
	LDH			1	1			1		4	6				8										
	Leucocytes						4	2			8				8										
	Lymphocytes											3			7		4								
	Monocytes			1			1				6				7		8								
	Neutrophils										1	3			8		8	1	2						
	Platelets									1			7		7		7			1					
	Potassium									5					7										
	Sex							2	1	4			8		7				1			1			
	Smoking							1							8		8						5		
	Sodium			1						3	4	1	3		8				3		1				

*Note:* All 23 variables are listed in both columns (parental nodes) and rows (child nodes). Connections highlighted on the expert‐elicited DAG are represented by green or orange fields below. A green field indicates the presence of the connection between the two variables on the expert‐elicited DAG, with the direction from the parental node to the child node (e.g., Age→ALAT). An orange square indicates that the connection was present on the expert‐elicited DAG but with the opposite direction (e.g., ALAT→Age). The number displayed inside fields indicates the count of DAGs learned from data (out of a total of 8) that agree on the specific connection. For example, five out of the eight DAGs learned from data agree on the connection Age→ALAT.

In general, the comparison between the expert‐elicited DAG and the DAGs learned from data revealed three trends. First, there was a general consensus between the expert‐elicited and DAGs learned from data concerning links going directly from LC to the other variables. Second, several links were only present on the expert‐elicited DAG, predominantly connections between several laboratory variables and age, smoking, and sex. Finally, the DAGs learned from data displayed several links not included in the expert‐elicited DAG. Some may reflect collinearity between variables, for example, the links between leucocytes and monocytes or leucocytes and neutrophils, both present in eight out of eight of the DAGs learned from data. Other links include CRP and leucocytes and CRP and LDH.

## 4 Discussion

### 4.1 Summary of Findings

In our experimental framework, BNs were employed to predict the risk of LC within a high‐risk population under examination for suspected LC over a decade in Southern Denmark. The dataset comprised 9940 patients with comprehensive information on 20 laboratory tests, age, gender, and smoking habits at the time of examination. We evaluated 16 distinct BN models, varying in the extent of missing data, the structure of the DAG utilized, and the method of discretization. We compared the performance to the previously presented DES model, developed on the same dataset.

Our findings indicate that AUCs remained consistent across the 16 models despite varying degrees of missing data, demonstrating robustness even with up to 30% missing values. The selection of DAGs derived from data versus expert‐elicited DAGs, as well as the choice between clinical and data‐driven discretization strategies, did not notably impact model performance.

Overall, the BNs demonstrated good calibration, despite a tendency to overestimate risk in higher‐risk intervals. Stage‐specific analysis revealed a disproportionate number of Stage I patients in lower risk intervals, whereas Stage II–IV patients were more evenly distributed across all risk intervals. Clinical utility analyses demonstrated a net benefit of utilizing BNs when predicted risk exceeded approximately 5%.

### 4.2 Interpretations

We observed that BNs show robustness across varying degrees of missing values. This was expected, since BNs have the ability to manage missing values by naturally incorporating them in a probabilistic framework. This ability is superior to imputation methods used in other ML models, which may become less reliable as the rate of missing data increases by potentially introducing bias and loses its generalizability [34, 39]. BNs also have a superior ability to handle certain structures of missing data; for example, if the data are missing at random (e.g., if information on smoking status was systematically missing in the younger population). This adaptability to missing data expands the pool of available data, which proves especially beneficial in screening scenarios where disease outcomes are relatively rare. Moreover, it facilitates collaborative efforts among hospitals on large‐scale projects without necessitating complete alignment of data collection methods. Given the growing trend of federated learning initiatives in both national and international healthcare projects, this feature of BNs emerges as exceedingly advantageous.

Our findings suggest that DAGs learned from data performed similarly to the expert‐drawn DAG, with overlapping 95% confidence intervals. While the expert‐drawn DAG incorporates domain knowledge and subjective judgment, the DAGs learned from data employ a systematic approach and statistical criteria to derive causal relationships from the data. Despite their differences, both approaches fundamentally rely on probabilistic inference among the included variables, which likely contributes to their similar performance. While expert DAGs are subjective and potentially inconsistent due to varying expert opinions, they can capture rare events deemed crucial by domain experts. Using a DAG learned from data offers the benefits of objectivity, consistency, and adaptability to new data compared to an expert DAG. However, it may be computationally intensive and unable to incorporate rare events not explicitly observed in the dataset. Additionally, a data‐driven approach cannot distinguish between causation and correlation, whereas an expert‐driven approach can. The equivalence in performance offers the mentioned benefit of using a DAG learned from data, which can also be easier to obtain and update than accessing expert knowledge in clinical everyday life.

There was no significant difference in the discretization strategies used, suggesting that the standard clinical reference intervals performed comparably to the data‐driven discretization method. While the data‐driven approach theoretically offers statistically justified cutoff points that could potentially be more accurate than arbitrary standard cutoffs, this distinction was not observed in this study. The similarity observed may stem from both methods using a similar number of bins, despite differences in their specific cutoff values. The fact that both strategies are equally effective is advantageous because clinicians are familiar with standard clinical reference intervals. This familiarity enhances the interpretability of DAGs using well‐established cutoff points that align with clinical guidelines and facilitate clinical decision‐making.

### 4.3 Strengths and Limitations

This study offers significant novelty through its large, cross‐regional dataset, which encompasses multiple hospital units. By utilizing the same dataset previously analyzed with traditional machine‐learning methods, we facilitate a direct comparison of results, enhancing the robustness of our findings. Additionally, the experimental design allows for the examination of performance across various rates of missing data while comparing different DAG structures and discretization strategies. This multifaceted approach provides valuable insights beyond mere comparisons with previous results.

Despite the mentioned strengths, our methodology also has limitations. As the complexity increases with high‐dimensional data containing a large number of variables, BNs become computationally burdensome and less practical for large datasets. Additionally, while BNs can handle high rates of missing data, they require high‐quality training data to accurately learn data structures.

The BNs developed in this study were based on a predefined cohort that has the strength of spanning over a decade and an entire region, encompassing multiple hospital sites and LC fast‐track clinics. This cohort comprises patients at high risk of LC, with both cases and controls referred for diagnostics in the LC fast‐track clinics. Consequently, LC and non‐LC patients share many similarities, posing challenges in discrimination compared to a standard case–control setup with healthy controls. While this setup can be viewed as advantageous due to the difficulty in detection, it also presents a limitation. Its applicability is restricted to other high‐risk cohorts with similar patient characteristics (e.g., a high rate of smokers both among LC and non‐LC patients) and LC incidence.

### 4.4 Clinical Implications and Future Perspectives

Using detection models capable of handling missing data carries clinical implications by potentially including patients with missing information, such as smoking status, in screening scenarios. This allows for personalized risk assessments for individuals who would otherwise be ineligible due to missing data.

Based on the decision curve analyses, no CT scan for patients with a risk level in the lowest 5% could be considered. However, this approach may be controversial, given that an LC risk of 5% is relatively high in screening scenarios, where most risk cutoffs typically range around 1.3%–1.5% [12, 40, 41]. However, the UKLS trial employed a risk cutoff of 4.5% (based on the LLpv2 risk model) for participant selection, marking the first randomized controlled trial to screen participants based on individual risk assessment tools [42]. Regardless of the exact cutoff values, individuals referred to LC fast‐track units usually present with symptoms suspicious of LC or with a detected nodule or infiltrate on a CT scan. Such factors raise concern, and individuals scoring 0%–5% in this BN model should not be disregarded, especially among patients referred for these reasons.

Consequently, the results of this current study are not directly applicable to the LC fast‐track cohort but require validation and potentially optimization on a lower‐risk population more akin to those eligible for LC screening. Future research is focusing on COPD outpatients, who are considered to be at a moderate risk of LC compared to the high‐risk fast‐track patients included in this current study. Currently, a Danish LC screening trial is in the planning phase. Depending on recruitment criteria, it may be pertinent to consider further development of BN risk models or validation on cohorts meeting these criteria.

In future research, we aim to develop a model using a small dataset but with a wide range of variables, including symptoms, comorbidity, and data from general practice. This model will then be validated on larger incomplete datasets containing “real” missing data, rather than artificially created missing data used in this study. If a BN model, trained on complete data, can reliably produce consistent results when applied to real‐world datasets with missing data, this represents a significant advantage in a screening scenario.

### 4.5 Comparison With Related Literature

As mentioned earlier, researchers in this study group have contributed to the development of a Dynamic Ensemble Selection (DES) ML model using the same study cohort as the current research. The DES model demonstrated moderate performance, achieving an AUC of 0.77 and a TPR of 24% at a TNR of 95%. In comparison, the most effective of the BNs developed in this study, model 2, exhibited a similar performance with an AUC of 75.6% (95% CI: 72.9%–78.3%) and a TPR of 20.6% (95% CI: 19.9%–21.3%) at a TNR of 95%. The improved performance of the DES model may stem from its capability to capture intricate relationships and manage large datasets with minimal assumptions about variable relationships. In contrast, BNs rely on stronger assumptions regarding probabilistic relationships. While advantageous for probabilistic reasoning and causal inference, these assumptions can constrain flexibility and accuracy in capturing complex data relationships. Both the DES and BN models showed similar calibration and clinical utility, with a positive net benefit above a risk cutoff of approximately 7% for DES compared to approximately 5% for the BN models. Comparing the performance of missing variable handling to DES is not feasible at this time. However, it would be intriguing to assess DES performance under conditions where the same rate of random missing values is introduced as in this study.

Several methodological differences pose challenges for direct comparisons with other prediction models. First, while the present model and the DES model were developed for LC detection, other models were crafted for LC prediction. For instance, the PLCOm2012 aimed to predict the 6‐year risk of LC with an AUC of 0.79 [43], while the MES model introduced by Gould et al. forecasted the 9–12 month risk of LC with an AUC of 0.86 [15]. Secondly, the PLCOm2012 relied on detailed questionnaires covering smoking duration, COPD, and cancer history, demanding considerable participant compliance. Lastly, both the MES model and PLCOm2012 were developed using large US population cohorts, characterized by a lower LC incidence compared to the high‐risk cohort in our study. Despite these disparities, if performance is compared, the BN may not strictly outperform other models, but its capability to handle missing data during parameter learning stands as a significant practical advantage. In contrast, both the MES and PLCOm2012 models require either relying on flawed imputation strategies or restricting datasets to nearly complete samples, as done with DES.

While numerous studies have evaluated the potential of liquid biopsies, such as circulating tumor DNA or microRNA, the volume of prediction models based on standard laboratory results remains sparse [44]. This may be attributed to inconclusive findings in association studies, as well as the limited complexity and data richness compared to liquid biopsy materials [45]. However, standard blood samples offer a cost‐effective approach and the ability to provide longitudinal information that facilitates monitoring changes over time [15, 46]. As research continues, there may be valuable opportunities to further explore standard blood samples, particularly when combined with emerging techniques to enhance predictive models for LC.

## 5 Conclusion

Our findings revealed that BN models exhibited consistent performance across varying levels of missing data, accommodating up to 30% missing values without significantly affecting discrimination. Notably, their performance closely paralleled that of a previously developed ML model (DES) on the same cohort, demonstrating comparable calibration and clinical utility.

The ability of BNs to manage missing data facilitates training on expansive datasets and simplifies integration into clinical settings, where data frequently appear sparse and incomplete. Consequently, BNs represent a promising pathway for future risk model development.

## Author Contributions

**Margrethe Bang Henriksen:** conceptualization (equal), data curation (lead), funding acquisition (lead), investigation (equal), methodology (supporting), project administration (equal), visualization (equal), writing – original draft (equal), writing – review and editing (equal). **Florian Van Daalen:** conceptualization (equal), formal analysis (lead), investigation (lead), methodology (lead), project administration (equal), resources (lead), software (lead), visualization (equal), writing – original draft (equal), writing – review and editing (equal). **Leonard Wee:** conceptualization (supporting), supervision (supporting), writing – review and editing (supporting). **Torben Frøstrup Hansen:** supervision (supporting), writing – review and editing (supporting). **Lars Henrik Jensen:** supervision (supporting), writing – review and editing (supporting). **Claus Lohman Brasen:** writing – review and editing (supporting). **Ole Hilberg:** supervision (supporting), writing – review and editing (supporting). **Inigo Bermejo:** conceptualization (supporting), supervision (supporting), writing – review and editing (supporting).

## Conflicts of Interest

The authors declare no conflicts of interest.

## Supporting information


Data S1.

## Data Availability

Data may be shared on reasonable request.
