# Supplementary material for: Lung Cancer Detection Using Bayesian Networks: A Retrospective Development and Validation Study on a Danish Population of High‐Risk Individuals
Source: Cancer Med. 2025 Jan 31;14(3):e70458. doi: 10.1002/cam4.70458 (PMC11783238; doi:10.1002/cam4.70458)
Supplement: Supplementary file 1 — Data S1. [file CAM4-14-e70458-s001.docx]

# Supplementary Appendix

Supplementary Table 1

|  | **Autogenerated discretization**  **based on minimum description length, 0% missing values*** | **Autogenerated discretization**  **based on minimum description length, 10% missing values*** | **Autogenerated discretization**  **based on minimum description length, 20% missing values*** | **Autogenerated discretization**  **based on minimum description length, 30% missing values*** |
| --- | --- | --- | --- | --- |
| P-ALAT, U/L | 15.5-24.5 | 15.5-30.5 | <17.5 | 12.5-16.5 |
| P-Albumin, g/L | <41.5 | <41.5 | <41.5 | <41.5 |
| P-Amylase (pancreatic), U/L | ALL | ALL | ALL | ALL |
| P-Alkaline phosphatase | <84.5 | <84.5 | <84.5 | <83.5 |
| B-Basophils, 10^9^/L | <0.045 | <0.045 | <0.045 | <0.045 |
| P-Bilirubin-total, **μ**mol/L | <7.5 | <7.5 | <7.5 | ALL |
| P-CRP, mg/L | 3.05-13.5 | 3.05-11.5 | 3.05-13.5 | <9.75 |
| Total Calcium, mmol/L | <2.395 | <2.395 | <2.395 | <2.395 |
| B-Eosinophils, 10^9^/L | <0.095 | <0.165 | <0.165 | <0.095 |
| B-Hemoglobin, mmol/L | 8.05-9.25 | 8.05-9.25 | 8.05-9.25 | 8.05-9.25 |
| P-INR | <0.795 | <0.795 | ALL | ALL |
| P-Potassium, mmol/L | ALL | <3.15 | ALL | ALL |
| P-Creatinine, mmol/L | <68.5 | 47.5-68.5 | <68.5 | <68.5 |
| P-LDH, U/L | 187.5-212.5-275.5-417.5 | 180.5-212.5-289.5-417.5 | 187.5-224.5-275.5-595 | 186.5-212.5-265.5-417.5 |
| B-Leucocytes, 10^9^/L | 6.165-7.855-11.15 | 6.205-7.855-11.15 | 6.105-7.855-11.15 | 6.275-7.855-11.15 |
| B-Lymphocytes, 10^9^/L | ALL | ALL | ALL | ALL |
| B-Monocytes, 10^9^/L | <0.745 | 0.705 | <0.745 | <0.705 |
| P-Sodium, mmol/L | 135.5-138.5 | 135.5-138.5 | 135.5-138.5 | 135.5-138.5 |
| B-Neutrophils, 10^9^/L | 3.485-5.065-6.935 | 3.485-5.065-6.875 | 3.555-5.065-6.935 | 3.475-5.065-6.905 |
| B-Platelets, 10^9^/L | 251.5-342.5 | 263.5-341.5 | 342.5 | 241.5-351.5 |

Comparison between standard discretization values derived from clinical guidelines' reference intervals and autogenerated discretization values using minimum description length, illustrated for data with missing values for 0%, 10%, 20% and 30% of the dataset. The numbers in each column indicate the threshold values for binning. "All" denotes the absence of cutoff values, resulting in a single bin encompassing all data points.

Supplementary Figure 1

Receiver Operating Characteristic (ROC) curves of the four levels of missing values. All models were based on DAGs learned from data with clinical discretization.


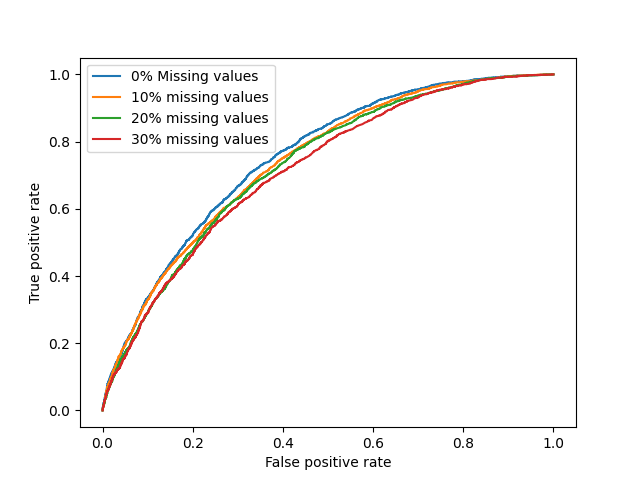


Supplementary Figure 2

Receiver Operating Characteristic (ROC) curves of the four levels of missing values. All models were based on expert-elicited DAGs with clinical discretization.


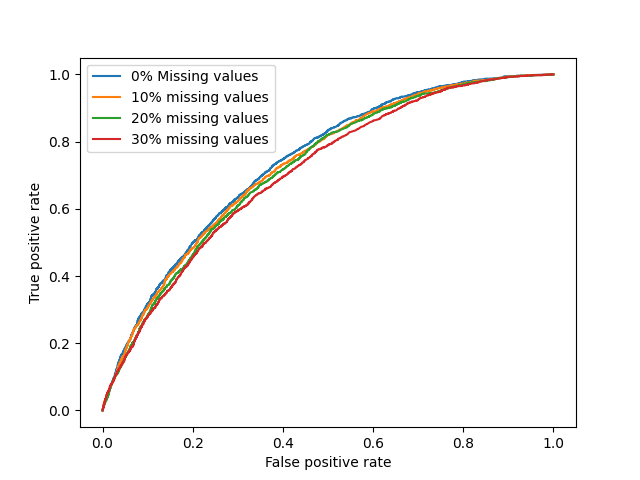


Supplementary Figure 3

Receiver Operating Characteristic (ROC) curves of the four levels of missing values. All models were based on expert-elicited DAGs with data driven discretization.


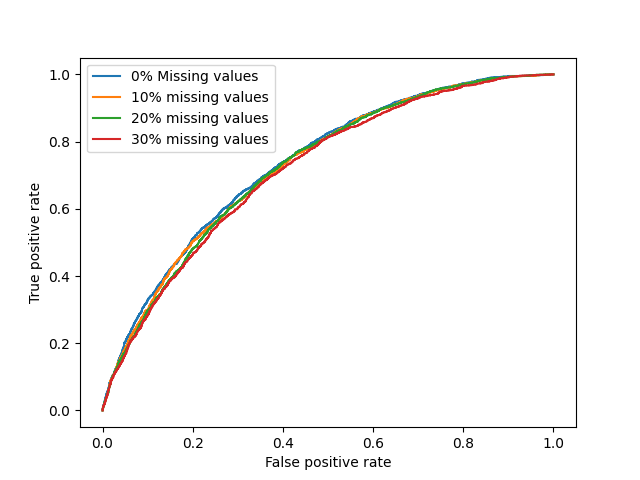


Supplementary Figure 4

The eight directed acyclic graphs (DAGs) learned from data with different degrees of missing values and different discretization strategies. All DAGs display the interconnections between the outcome lung cancer (LC) and all other variables. Images were rendered using OPEN Markov[1]


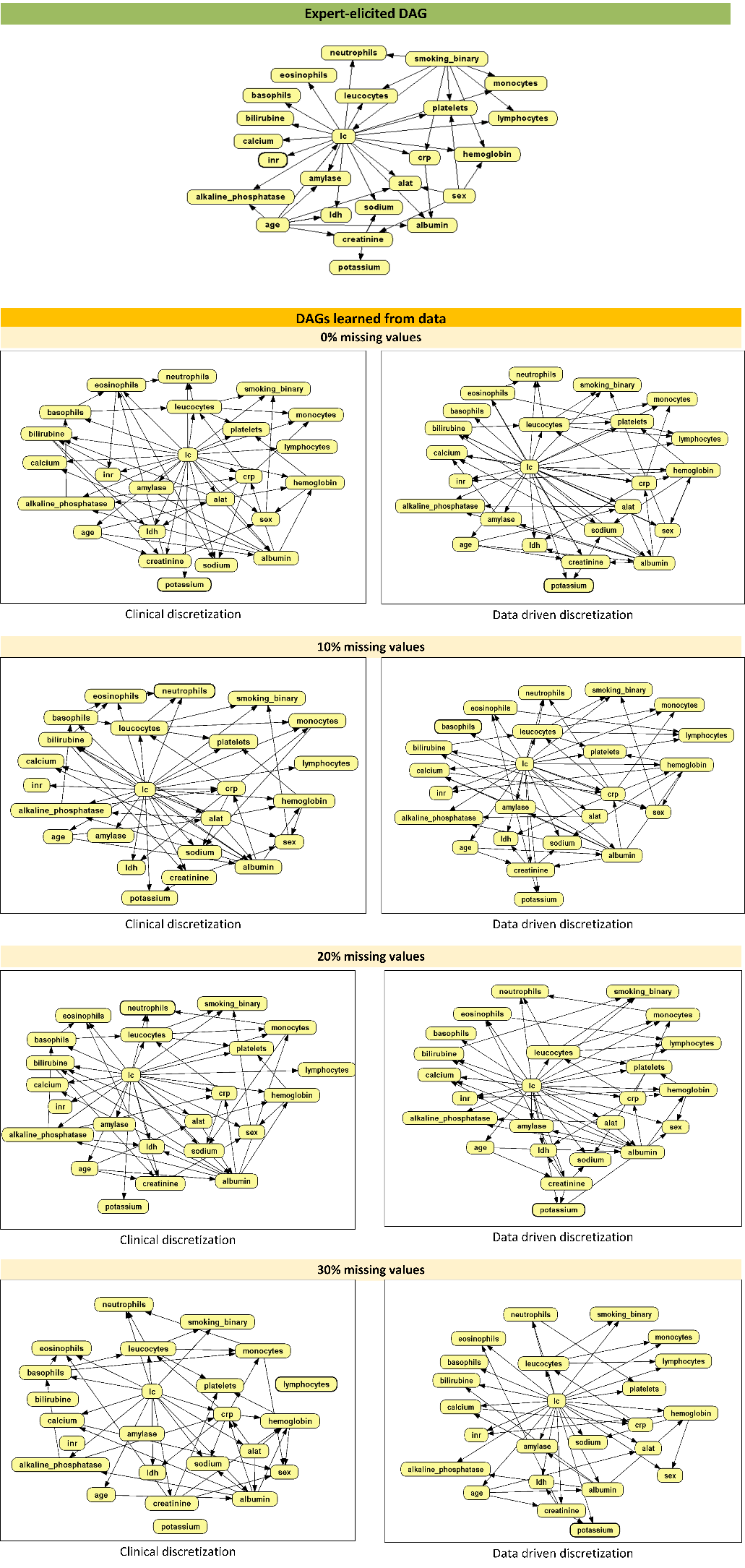


## References

1. Arias M, Pérez-Mart\’\in J, Luque M, D\’\iez FJ. OpenMarkov, an Open-Source Tool for Probabilistic Graphical Models. *IJCAI* 2019. p. 6485–6487.
